# Supplementary material for: Population genetic simulation study of power in association testing across genetic architectures and study designs
Source: Genet Epidemiol. 2019 Oct 6;44(1):90–103. doi: 10.1002/gepi.22264 (PMC6980249; doi:10.1002/gepi.22264)
Supplement: Supplementary file 1 — Supplementary information [file GEPI-44-90-s001.docx]

**Supplemental Information**

Population genetic simulation study of power in association testing across genetic architectures and study designs

Dominic Ming Hay Tong^1^, Ryan D. Hernandez^1,2,3^

^1^University of California, Berkeley – University of California, San Francisco Graduate Program in Bioengineering

^2^Department of Bioengineering and Therapeutic Sciences, University of California, San Francisco

^3^Department of Human Genetics, McGill University, Montreal, QC, Canada

Figure S1. The statistical power of SKAT-O under an imputed study design does not change when the reference panel size is increased. Each point represents 10 independent simulations of a genetic architecture. The larger square point in the middle of each distribution indicates the mean SKAT-O power under each reference panel size.

Figure S2.  A global overview of the statistical power of a burden test (KBAC), a variance-component test (SKAT), a combined test (SKAT-O), and a single-variant logistic regression test (GWAS) for all parameters shown in Table 1 using 50,000 African individuals simulated under an Out-of-Africa demographic model. Each point represents a genetic architecture tested with 10 independent simulations under the RVAT indicated; lines connect the same simulated parameters across RVATs to show that, generally speaking, the rank of statistical power is preserved across RVATs.

Figure S3. The statistical power of SKAT-O across different sampling strategies (columns) and across different sequencing methods (rows), as a function of the proportion of genetic variance explained by that genetic architecture at MAF=1%. Each point represents 20 independent simulations of 10 or 100 causal loci of 10kb each across a 5Mb simulated region for a given genetic architecture for a 50,000 individual African population.

Figure S4. The statistical power of GWAS across different sampling strategies (columns) and across different sequencing methods (rows), as a function of the proportion of genetic variance explained by that genetic architecture at MAF=1%. Each point represents 20 independent simulations of 10 or 100 causal loci of 10kb each across a 5Mb simulated region for a given genetic architecture for a 50,000 individual African population.

Figure S5. The mean absolute effect sizes for alleles at different minor allele frequency (MAF) for various parameter combinations (from Table 1, main text).
